# Strengthening contact tracing capacity of pulmonary tuberculosis patients in Enugu, southeast Nigeria: a targeted and focused health education intervention study

**DOI:** 10.1186/1471-2458-14-1175

**Published:** 2014-11-18

**Authors:** Osa-eloka Christiandolus Ekwueme, Babatunde I Omotowo, Kennedy Kenechukwu Agwuna

**Affiliations:** Department of Community Medicine, University of Nigeria, Enugu Campus, PMB 01129, Enugu, Nigeria; Department of Radiology, University of Nigeria, PMB 01129, Enugu, Nigeria

**Keywords:** Tuberculosis, Contact tracing, Health education, Knowledge, Nigeria

## Abstract

**Background:**

Nigeria ranks 10 out of the 22 countries in the world with the highest TB burden. Contact tracing enhances case finding and increases the probability of cure. The purpose of the study is to improve the contact tracing skills of tuberculosis patients at the major TB centre in Enugu State, Nigeria.

**Methods:**

The study is an educational intervention with a study and a control groups selected using multi-stage sampling techniques. A calculated sample size of 190 patients was used for each group. The instrument was a pre-tested semi-structured interviewer administered questionnaire. Data entry and analysis was done using Epi-info version 3.3.2. Chi-square test and student t –test were used at p < 0.05 level of significance and 95 percent confidence interval.

**Results:**

Awareness of contact tracing at baseline and post intervention were respectively 18.2% and 85.2% (X^2^ = 158.4, DF = 1, p = 0.000; CI: 15.8-82.2) for the study group; 18.4% and 26.0% (X^2^ = 3.31, DF = 1, p = 0.069; CI: -9.9-24.7) for the control group. Knowledge that contact tracing involve bringing all household contacts of TB patients for screening was 79 (44.9%) and 33 (19.2%) for the study and control groups at baseline (X^2^ = 26.32, p = 0.000; CI: 7.2-44.1), but 151 (85.8%) and 36(20.9%) for the same at post-intervention (X^2^ = 147.22, p = 0.000; CI: 49.3-80.1). At baseline, only 5 (2.8%) of the study and 6(3.5%) of the control groups ( X^2^ = 0.12, p = 0.730; CI: -14.2-12.8 ) brought two or more contacts for screening. At post-intervention, the figure rose to 114 (64.8%) and 9 (5.2%) (X^2^ = 134.94, p = 0.000; CI: 44.3-74.9) for the study and control groups respectively. Over 50% of the contacts brought for screening were less than 10 years; 31 (18.3%) at baseline to 138 (81.7%) post-intervention in the study group (CI: 47.6-79.2), and 26 (35.1%) to 38 (51.4%) for the control group (X^2^ = 12.472, p = 0.000; CI: 0.1 -32.5).

**Conclusion:**

Intensive planned health education intervention has been used to improve the contact tracing skills of the TB patients in a major TB centre in Enugu State, Nigeria. Further training and re-training of TB patients on contact tracing is highly recommended.

## Background

Tuberculosis (TB) remains a major global health problem. It is a chronic, infectious disease caused by bacteria generally referred to as ‘Mycobacterium tuberculosis complex. The most important source of infection is an untreated pulmonary TB (PTB) patient, which releases thousands of tiny droplet nuclei when he or she coughs, spits, or sneezes. Transmission is airborne through inhalation of the released tubercle bacilli [1]. Tuberculosis causes ill-health among millions of people each year, and ranks as the second leading cause of death from an infectious disease worldwide, after the human immunodeficiency virus (HIV). The latest estimate is that there were almost 9 million new cases in 2011 and 1.4 million TB deaths [2]. Out of these 9 million active cases, 5% occur in the developed countries, and 95% in developing countries [3, 4].

Nigeria is among the nations of the world with the highest TB burden, worsened by the challenges of HIV co-infection, cases of drug-resistant TB (DR TB), and TB in children which accounts for 10-20% of all TB cases [1]. According to the World Health Organization (WHO) reports on global TB, Nigeria ranks 10 out of the 22 countries in the world with TB prevalence, which has contributed 80 per cent of the estimated 9 million new cases in the world [1, 5]. Nigeria established its National TB and Leprosy Control Program (NTBLCP) in 1989 and Directly Observed Treatment Short Course (DOTS) strategy in 1994 [6]. In line with the Millennium Development Goals (MDGs), endorsed by the Stop TB partnership, the targets of Nigeria National TB program goal is to detect 70% of infectious TB cases, cure at least 85% of these cases by 2005, reduce TB prevalence and death rates by 50% relative to 1990 by 2015, and eliminate TB as a public health problem. This means to have a prevalence of less than one case per million population by 2050 [1, 7]. However, the case detection rate for new and relapsed cases in Nigeria in 2011 was 45%, 26% and 96% at best lower and upper boundaries respectively [2]. Treatment success rate for new smear positive cases in a cohort study in 2010 was 84% [2]. However, successes achieved were attributed not only to DOTS, but also to other TB programme elements such as health education, reminders, contact tracing, screening and defaulters tracing [8–10].

Contact tracing, the evaluation of persons who have been in contact with patients having tuberculosis is an important component of tuberculosis control [11]. Contact tracing can be active or passive. In active contact tracing, health workers regularly visit the family of each TB case and keep a contact register. Passive contact tracing, which happens at the TB clinics, requires adequate education of each TB patient, in order to stimulate him or her to present any family member with suspect symptoms to the health service for screening. Contact tracing enhances case finding and thereby increases the probability of cure [11]. Furthermore, contact tracing makes it possible to identify patients in initial stages of the disease, when infectivity and morbidity are still low [12].

Contacts of TB patients are at high risk of acquiring either active TB or TB infection, depending on factors such as sources of infection, type of contact, and environmental characteristics [13]. Therefore, the search for infected subjects among relative of patients and household members with infectious tuberculosis is the best method of preventing later development of disease in populations where the prevalence is low [13]. It is estimated that 2–3 persons would be infected by a smear positive case before its detection in developed countries, as against 4–5 persons in the developing countries because of higher number of close contacts [14]. It was observed in some studies that contacts of a tuberculosis patient are 10 to 60 times more likely to have the disease than the general population, and approximately 10-14% of all notified cases have been detected by contact screening [14]. Contact examination has a valuable impact on health education, and impresses on the family and community the infectious nature of the disease, the need for proper and regular treatment. This would result ultimately in greater adherence to treatment and improvement in cure rates [15].

Studies have shown that examination of household contacts, especially of known sputum-positive cases, is very effective in tuberculosis control. The study done on tuberculosis contact tracing among children and adolescents in Brazil, revealed that 92.4% of them had household contacts, and 66.5% of the contacts were the child’s parents. About 13.6% of TB was detected in this study, out of which 28% were asymptomatic. It was also reported in the same study that there was greater occurrence when the contact lived with more than one source of infection [16]. Another study done on the risk of active tuberculosis in adult household contacts of smear-positive pulmonary tuberculosis cases in Turkey recorded a detection rate of 5.4%. Rates of active TB were highest in the age groups 15–24 year (8.5%) and 25–34 year (6.5%). A similar household survey study in Malawi found that young children from households where one or both parents have smear-positive pulmonary tuberculosis are at increased risk of developing active and disseminated tuberculosis. It was observed from the same study that 40% of patients with smear-positive PTB had young children living at home, yet only 21% of the adult patients were informed about the need for childhood screening [17].

A survey study conducted by Centers for Disease Control and Prevention in United States between July 1996 and June 1997, showed that out of total 6,225 close contacts, 43% were household contacts, 18% were relatives not living in the household, 12% were co-workers, 9% were leisure contacts, and 18% were other types of contacts [18]. In the Netherland, a study on TB infection in children who are contacts of immigrant TB patients, reported that 2.5% of the contacts had TB, and that contact of sputum smear positive patients, significantly more often than not, had a positive tuberculin skin tests (25%), compared with the contacts of sputum smear-negative patients (7%) [13]. It has therefore being recommended by the World Health Organization that screening of the contacts should include at least, a thorough history, clinical examination, tuberculin test, chest X-ray and HIV test [19].

In view of its importance in TB management, despite the lack of resources for health care in the developing country like Nigeria, contact tracing should be considered as an important preventive measure to be established and regularly used for TB control purposes. Thus the need for this study, which is aimed at improving the contact tracing knowledge and skills of TB patients to bring their contacts for screening at the major TB centre in Enugu State, Nigeria. The following null and alternative hypotheses are proposed for the study:

### Null hypothesis

1. There is no significant difference between the study and the control TB patients on the knowledge of contact tracing before and after health education intervention.
2. There is no significance difference on the number of the study and the control TB patients that brought TB contacts for screening before and after health education intervention.

#### Alternative hypothesis

3. There is a significant difference between the study and the control TB patients on the knowledge of contact tracing before and after health education intervention.
4. There is a significant difference on the number of the study and the control TB patients that brought TB contacts for screening before and after health education intervention.

## Methods

### Study sites

The study was carried out in Enugu and Ebonyi States, two of the five southeastern states that made up the thirty-six states of Nigeria. Enugu and Ebonyi States respectively has a population of 3,140,471 and of 2,852,832 people by 2006 population census. The states are mainly populated by people of Igbo extraction, with few non indigenes migrating from the Yoruba, Hausa, Ijaw and Isoko and other ethnic groups of Nigeria. Civil service and subsistence farming are the two predominant occupations in these states.

The intervention study site, the Chest Unit of University of Nigeria Teaching Hospital (UNTH) Enugu is the biggest directly observed treatment short course (DOTS) center among the 56 DOTS centers in Enugu state. The unit registers an average of 20 new smear positive patients each month, and has an annual turnover of 250 – 300 new patients. At the center, the outpatient TB clinic runs thrice in a week, Mondays, Wednesdays and Fridays.

The control site, Mile 4 TB Center located in Abakaliki, is the largest out of the 15 TB centers in Ebonyi state. The unit has an annual turnover of 120 – 150 new patients. In this centre, outpatient TB clinic runs every day, Monday through Friday. The study and the control sites are located at a distance of 100 Kilometers apart and are in different states of Nigeria.

### Study design

The study was an educational intervention with a quasi-experimental design. There was a baseline study for the two groups which was followed by an educational intervention on the study group only and then a post-intervention study for the two groups. However, the control group was after the study given a health education on tuberculosis control mechanism and contact tracing. The aim is primarily to give them the benefits of the health education intervention, and to position them to contribute to the national TB control efforts of the Federal Republic of Nigeria.

### Participants

**Figure 1:**
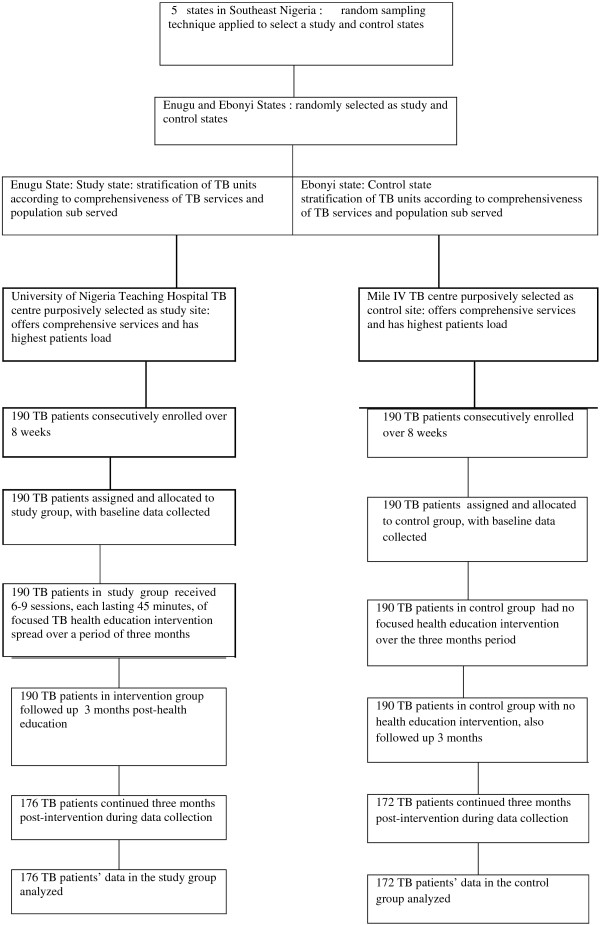
**Participants’ flow chart.**

The participants are sputum smear positive TB patients accessing treatments at the selected TB centers in Enugu and Ebonyi States, southeast, Nigeria. A multistage sampling method was used. The first stage was the selection of Enugu and Ebonyi states from the five states in the southeastern Nigeria using simple random sampling technique. In each of the selected states, the list of TB units was compiled using the Directly Observed Treatment Short Course TB units Directory (DOTS Directory). The Units were stratified according to services offered, and the centers that offer both laboratory and treatments services were enlisted. From the enlisted TB centers in each of the states, a center which sub-serves the greatest number of patients was selected. In this case, UNTH Chest Unit Enugu and Mile 4 Hospital Abakaliki were purposively selected. At the level of patient selection, each of the centers was visited during their clinic days, Monday, Wednesday and Friday for UNTH and Monday through Friday for Mile 4 TB Units. All and only smear positive TB cases that were present during the days of visit were consecutively recruited for the study after given and receiving an informed oral consent. The recruitment was done at each of the centers during their clinic days until the desired sample size was obtained. The recruitment exercise lasted for approximately eight weeks Figure 1.

A calculated sample size of 190 patients each for the study and control groups was used for the study, using a formulae for comparison of proportions at a power of 80% with a prevalence value of contact tracing among children and adolescent without education put at 13.6% [20].

### Measures to minimize potential bias inherent to non-randomization studies

Stratification of the TB centers according to completeness of services offered and the patients load was done before selection of the study and control units. Only sputum smear positive TB patients who had started treatment at least one month prior to the study were included in the study. This measure minimizes any differences in the treatment experiences among the TB patients and reduces potential differences in attrition rate. Matching of TB patients was done at the group levels and participants with similar socio-demographic variables were selected. Group level matching was done to minimize resource and logistic cost implications. The process ensured that the TB patients have similar characteristics at baseline. Strategies were taken to confirm that all the TB patients in the study and the control groups have had exposure to the routine health education at their TB centres. Both groups have similar environmental and geographical exposure having been selected from the same southeast region of the country, and have the same ethnic background.

### Interventions

In the Nigerian National tuberculosis and leprosy control programme guideline, the health workers are required to give health education to all the patients at the time of diagnosis and at the time of discharge [3]. At the time of diagnosis, the health education is expected to focus mainly on the aetiology, diagnosis including informing the patient his sputum test result and treatment modalities. The health worker is also expected to inform the TB patients the need to invite their contacts to the health facility to be screened for tuberculosis. At the discharge, the health worker should inform the patient the outcome of his or her treatment, the need to return for check up, and to bring along their contacts to the TB clinic for screening. In practice and from experience in our setting, the health education is basically an informal sharing of health information, done passively without getting the patients involved in the education process.

In this study, the health education intervention was intensive and innovative [21]. It was done on a daily bases for the sputum smear positive patients throughout the period of the study, bearing in mind that their contacts are the high priority groups. The new smear positive TB patients on directly observed treatment short course (DOTS) chemotherapy that comes daily to take their drugs were thoroughly given health education on TB contact tracing, motivated to change negative behaviours and attitudes inimical to the desired goal of convincingly and persuasively bringing their contacts for screening. The key aspects of the planned behaviour conceptual frame work are based on Ajzen’s theory of planned behaviour and Bandura social cognitive (learning) theory aimed at changing some personal factors that might negatively affect the expected outcome. This is a form of modified passive health education intervention on contact tracing among TB patients, involving their active participation, and behaviour change which is different from the routine health education on passive contact tracing. Although the environment of the health education is in a passive setting, the process of informing, reaching and getting the TB contacts come for screening takes the form of active contact tracing. The first health education intervention was given to the smear positive TB patients at their first visit after recruitment, being also the day diagnosis were made. This is to avoid missed opportunity of giving this all important aspect of TB control knowledge to these patients. The health educations were subsequently given in groups as the first thing in the morning before commencement of consultation with the doctors.

Sputum smear positive TB patients recruited in the study but who have completed their DOTS were given weekly appointments for the purpose of this study. This group of patients received a minimum of six to nine health education sessions that lasted for at least 45 minutes. The patients still on their intensive phase of DOTS chemotherapy had more than nine sessions of the health education since they attend clinic daily for their drugs. After each health education session, fifteen minutes were devoted for questions and answers to enable the TB patients to get clarifications to gray areas and intriguing concerns. A phone call serving as a reminder of the appointment day was usually given to each patient a day before through their mobile cell phones or that of their next of kin, family member or treatment supporter as supplied by the patients. Attendance register was opened to monitor the number of health education sessions attended by each patient.

The study was undertaken within the months of February, 2012, to June, 2013. Recruitments of the TB participants took place between the months June to August, 2012, and lasted for eight weeks. Health education intervention was implemented over a period of three months starting from September to November, 2012. Post-intervention and follow up of the TB participants was from January, 2013, to March, 2013. Data analysis, write up and reporting lasted for three months. The intervention was carried out only at the study site, the UNTH Chest clinic. There was no intervention at the control site, Mile 4 Hospital, Abakaliki. The targeted and focused health education intervention laid emphasis on the meaning, importance of contact tracing on the control of tuberculosis, and mechanisms of contact tracing. Other topics covered during the health education intervention include the causes, signs and symptoms of Tuberculosis; sources of TB infection and mode of transmission; and the factors that affect the development and spread of tuberculosis [22]. The training was done by the researchers and assisted by the research assistants. Formal routine health talk done at the time of diagnosis and discharge on TB disease at the control center, Mile 4 Hospital, Abakaliki were allowed unabated during the period of intervention at the study site. This is to avoid unnecessary questions and inquisitives on the side of the control group and their health workers who were kept blind as to the ongoing intervention at the study site.

### Data collection and analysis

The data collection was carried out concurrently in the two TB centers. A semi-structured questionnaire was used. The questionnaire was interviewer administered. The questionnaire was pre-tested in a TB unit that was not selected for the study. Three trained research assistants administered the questionnaire in each of the TB center. Information elicited included the socio-demographic characteristics, knowledge about tuberculosis including the causes, signs and symptoms, risk factors of pulmonary tuberculosis, transmission and control, contact tracing mechanisms and contact characteristics. The primary outcome measures are the number of TB patients that brought their contacts for TB screening, and the actual number of contacts brought for screening post-intervention. The secondary outcome measure is the knowledge difference on contact tracing between the study and control groups post-intervention.

Data entry and analysis was done using Statistical Package for Social Sciences (SPSS) soft ware version 11 and Epi-info version 3.3.2. Selected variables were compared between the study and the control groups, and where necessary using Chi-square test, and student T –test set at p < 0.05 level of significance, and 95 percent confidence interval. Ethical approval was obtained from the Ethics Committee of the University of Nigeria Teaching Hospital, Enugu. Individual informed oral consent was obtained prior to the recruitment and health education intervention.

## Results

One hundred and ninety (190) sputum smear positive tuberculosis patients each were enrolled in the study and control groups. Post-intervention, 176 and 172 respectively were studied giving an attrition rate of 7.4% and 9.5%. Socio-demographic characteristics of the groups did not differ significantly (p > 0.05) Table 1. Awareness of contact tracing differs in the study and control groups by 0.2% and 59.2% at baseline and post-intervention respectively.

**Table 1:** **Socio-demographic characteristics of respondents**

Socio-demographic characteristics	Study group	Control group	Ch-square	P value	95% CI
	No (%)	No (%)			
	N = 176	N =172			
Age: mean ± SD	31.7 ± 10.4	33.4 ± 11.0	t =1.48	0.139	
Gender:					
Male	89(50.6)	81(47.1)	0.420	0.517	-24.1 - 17.1
Female	87(49.4)	91(52.9)			-24.2 - 17.1
Occupation:					
Student	39(22.2)	32(18.6)	2.450	0.784	-13.0 - 20.2
Trader	31(17.6)	28(16.3)			-14.2 -16.8
Farmer	21(11.9)	24(14.0)			11.7 - 15.9
Civil servant	18(10.2)	20(11.6)			11.5 - 14.3
Artisan	9(5.1)	5(2.9)			-5.8 -10.3
Others	58(33.0)	63(36.6)			16.6 -22.6
Marital status:					
Ever married	80(45.5)	90(52.3)	1.640	1.998	13.7 - 27.3
Never married	96(54.5)	82(47.7)			-13.7 - 27.3
Education:					
None	19(10.8)	28(16.3)	2.530	0.469	8.6 - 19.6
Primary	36(20.5)	30(17.4)			-13.0 - 19.2
Secondary	74(42.0)	72(41.9)			-20.2 - 20.4
Tertiary	47(26.7)	42(24.4)			-15.7 - 20.3
Total	176(100.0)	172(100.0)			

Table 2 shows that at baseline, less than 50% of the TB patients in the study (76) and the control (55) groups knew that the causative agent of TB is a bacterium. Again less than 50% of the patients in either group could identify correctly any of the routes of transmission of TB, except for the study group, where 70.5% identified transmission by close contact with smear positive case. Among the study group, 65.3% and 62.5% respectively knew that crowded conditions and poor housing are risk factors of TB transmission, against 21.5% and 22.1% in the control group. Over 70% of the study group additionally knew each of the various methods of TB control compared to less than 50% in the control group.

**Table 2:** **Knowledge of the tuberculosis disease by the TB patients at baseline**

Knowledge about TB disease	Study group	Control group	Chi-square	P-value	95% CI
	N = 176(%)	N = 172 (%)			
**Causes:**					
Bacteria	76(43.2)	55(32.0)	4.65	0.031	-8.6 - 31.0
Evil Spirit	20(11.4)	13(7.6)	1.47	0.226	-8.2 - 15.8
Food poisoning	39(22.4	18(10.5)	8.69	0.003	-3.2 - 26.9
**Mode of transmission:**					
Airborne	77(43.8)	55(32.0)	5.12	0.024	-8.0 - 31.6
Patients to contact	124(70.5)	68(39.5)	33.63	0.000	11 - 50.5
Consume infected cow milk	71(40.3)	26(15.1)	27.53	0.000	7.5 - 42.9
Waterborne	32(18.2)	52(30.2)	6.90	0.001	5.5 - 29.5
food borne	33(18.8)	28(16.3)	0.37	0.544	-13.2 - 18.2
**Risk factors:**					
Crowded conditions	111(65.3)	37(21.5)	61.46	0.000	25.5 - 62.1
Poor housing	110(62.5)	38(22.1)	58.11	0.000	21.9 - 58.9
Smoking	107(60.8)	77(44.8)	8.97	0.003	-4.3 - 36.3
Alcohol	87(49.4)	77(44.8)	0.76	0.383	-15.9 - 25.1
Malnutrition	85(48.3)	37(21.5)	27.41	0.000	7.98 - 45.6
Poverty	66(37.5)	50(29.1)	2.78	0.095	-10.9 - 27.7
Age	36(20.5)	30(17.4)	0.51	0.474	-13.0 - 19.2
**Methods of TB control:**					
Cough etiquette	153(86.9)	80(46.5)	64.23	0.000	22.8 - 57.9
Early diagnosis and treatment	130(73.9)	83(48.3)	24.02	0. 000	7.2 - 44.9
Increasing natural ventilation	134(76.1)	65(37.8)	52.24	0.000	19.5 -57.1
Nutritional improvement	132(75.0)	73(42.4)	38.09	0.000	13.5 - 51.8
Immunization with BCG	123(69.9)	77(44.8)	22.46	0.000	5.4 -44.8

Table 3 shows that 79 (44.9%) and 33 (19.2%) of the TB patients in the study and control groups at baseline knew that their household members should be brought for screening. Post-intervention, the study group knowledge increased significantly to 151 (85.8%), while the control was 36 (20.9%). The differences observed in the knowledge of contact tracing between the study and the control groups at baseline and post-intervention was statistically significant (79 vs.33, X2 = 26.32, p = 0.000, 95% CI 7.3-44.2; 151 vs. 36, X2 = 147.22, p = 0.000, 95% CI 49.3-80.5). The study group also had a statistically significant difference in the knowledge of contact tracing at baseline and post-intervention as shown in Table 4 (79 vs.151, X2 = 65.03, p = 0.000, 95% CI 23.3-58.5).

**Table 3:** **Knowledge of contact tracing among study and the control TB patients**

Knowledge	Baseline		Chi-square	P-value	Post- intervention		Chi-square	P-value
	Study group	Control group			Study group	Control group		
	N = 176(%)	N = 172(%)			N = 176(%)	N = 172(%)		
**Meaning of contact tracing:**								
Screen all household members	79(44.9)	33(19.2)	26.32	0.000	151(85.8)	36(20.9)	147.22	0.000
**95% CI**	7.3-44.2				49.3-80.5			
Screen only members who are coughing	107(60.8)	109(63.4)	0.25	0.620	4(2.3)	95(55.2)	119.86	0.000
**95% CI**	17.4-22.5				-37.7-68.1			
Screen only household coughing out blood	114(64.8)	124(72.1)	2.16	0.142	9(5.1)	119(69.2)	153.58	0.000
**95% CI**	11.8-26.4				-49.2-79.1

**Table 4:** **Within groups comparison of knowledge of contact tracing among TB patients**

Knowledge	Study group		Chi-square	P-value	Control group		Chi-square	P-value
	Baseline	Post-intervention			Baseline	Post-intervention		
	N = 176(%)	N = 176(%)			N = 172(%)	N = 172(%)		
**Meaning of contact tracing:**								
Screen all household members	79(44.9)	151(85.8)	65.03	0.000	33(19.2)	36(20.9)	0.16	0.686
**95% CI**		23.3-58.5			-14.9-18.3			
Screen only members who are coughing	107(60.8)	4(2.3)	139.60	0.000	109(63.4)	95(55.2)	2.36	0.124
**95% CI**	-43.7-73.3				12.1-28.5			
Screen only household coughing out blood	114(64.8)	9(5.1)	137.78	0.000	124(72.1)	119(69.2)	0.35	0.554
**95% CI**	-44.5-74.9				15.9 -21.8			

Table 5 shows that over 80% of the TB patients in the study and control groups had never brought any contacts for screening at baseline. Post-intervention, the number of patients in the study group that brought two or more contacts for screening rose to the significant level of 64.8% against 5.2% in the control group (p = 0.000). The total number of contacts brought for screening at baseline and post-intervention as shown in Table 6 by the study and control groups were 31 and 138; 26 and 38 respectively (X^2^ = 12.472, p = 0.000, 4.13E-04). Most of the contacts were below 10 years of age.

**Table 5:** **Practice of contact tracing by the study and control TB patients**

Practice	Baseline		Chi-square	P-value	Post- intervention		Chi-square	P-value
	Study group	Control group			Study group	Control group		
	N = 176(%)	N = 172(%)			N = 176(%)	N = 172(%)		
**Number of contacts brought for screening:**								
None	153(86.9)	154(89.5)	0.57	0.451	42(23.9)	142(82.6)	120.27	0.000
**95% CI**	10.7-15.9				54.4-63.0			
One	18(10.2)			0.281	20(11.4)	21(12.2)	0.02	0.883
**95% CI**	8.4-14.7	12(7.0)	1.17		14.1-12.5			
Two or more	5(2.8)	6(3.5))	0.12	0.730	114(64.8)	9(5.2)	134.94	0.000
**95% CI**	12.8-14.2				44.3-74.9

**Table 6:** **Socio-demographic characteristics of contacts brought for screening by TB patients**

Socio-demographics characteristics	Study group		Control group	
	Baseline post-intervention		Baseline post-intervention	
	N = 31 (%) N = 138(%) 95% CI		N = 26 (%) N = 38(%) 95% CI	
Age (years):				
<10	15(48.4)	76(55.1) -44.8-31.4	18(69.2)	24(63.2) -39.9-51.9
10-19	9(29.0)	20(14.5) -18.9-47.9	4(15.4)	7(18.4) -39.4-33.4
20-39	5(16.1)	24(17.4) -29.5-26.9	4(15.4)	4(10.5) -28.4-38.2
40+	2(6.5)	18(13.0) -27.7-14.1	0(0.0)	3(7.9) 45.3-61.1
Gender:				
Male	13(41.9)	78(56.9) -52.9-22.9	12(46.2)	18(47.4) -50-47.6
Female	18(58.1)	60(43.5) -23.1.52.3	14(53.8	20(52.6) -47.6-50.0
Marital status:				
Single	27(87.1)	116(84.1) -23-29	22(84.6)	32(84.2) -35-35.8
Married	4(12.9)	22(15.9) -29-23	4(15.4)	6(15.8) -35-35.8
Occupation:				
Student	27(87.1)	129(93.5) -47-34.2	21(87.5)	33(86.8) -31.9-33.8
Others	4(12.9)	9(6.5) - 18.1-30.9	3(12.5)	5(13.2) -33.3-31.9
Education:				
None	2(6.5)	11(8.0) -17.7-20.7	2(7.7)	3(7.9) -26.0-26.4
Primary	18(58.1)	79(57.2) -36.8-38	18(69.2)	24(63.2) -40-52
Secondary	9(29.0)	42(30.4) -36.1-33.3	3(11.5)	7(18.4) -41-27.2
Tertiary	2(6.5)	6(4.3) -16.1-20.5	3(11.5)	4(10.5) -29.5-31.5
Living with patient:				
Yes	30(96.8)	120(87.0) -6.6-26.2	23(88.5)	32(84.2) -28-37
No	1(3.2)	18(13.0) -28-8.4	3(11.5)	6(15.8 -37-28
Relationship with patient:				
Children/siblings	29(93.5)	132(95.7) -20-16.1	19(73.1)	26(68.4) 39.5-48.9
Friends/others	2(6.5)	6(4.3) -16.1-20.5	7(26.9)	12(31.6) - 48.9-39.5
Total	31(100.0)	138 (100.0)	26 (100.0)	38 (100.0)

Chi-square of the total contacts at baseline and post-intervention for the study and control groups: X^2^ = 12.472, df = 2, p = 0.000 [4.13E-04].

Table 7 shows that fever and cough were the predominant symptoms presented by the contacts. Prevalence of tuberculosis among the contacts of the study and control groups at baseline and post-intervention were 19.4% and 13.7%; and 26.9% and 28.9% respectively as shown in Table 8.

**Table 7:** **Clinical features of contacts brought for screening at the study and control centers**

Clinical features	Baseline (six months pre-intervention)	Post-intervention (six months period of intervention)	
	N = 57 (%)	N = 176 (%)	95% CI
Cough	7(12.3)	15(8.5)	-14.8-22.4
Fever	7(12.3)	12 ( 6.8)	-12.7-23.7
Night sweats	3(5.3)	5 (2.8)	-10.4-14.9
Weight loss	8(14.0)	11 (6.3)	-11.3-26.7
Haemoptysis	1(1.8)	5 (2.8)	-7.3-9.3
Tuberculin test:			
Positive	18(31.6)	45 (25.6)	-20.8-32.8
Negative	39(68.4	131(74.4)	-32.8-20.8
Chest X-ray report:			
Positive	7(12.3)	13(7.4)	-31.6-23.3
Negative	50(87.7)	163 (92.6)	-23.3-13.5
Sputum AFB result:			
Positive	5(8.8)	11(6.3)	-13.6-18.5
Negative	52(91.2)	165(93.8)	-18.6-13.4
Total	57(100.0)	176(100.0)

**Table 8:** **Proportion of contacts diagnosed and treated as case of tuberculosis**

TB diagnosed	Baseline	Post-intervention		Chi-Square	P value
	N = 57(%)	N = 176(%)	95% CI		
**Study contacts:**	N = 31	N = 138			
Yes	6 (19.4)	19 (13.7)	-23.8-35.2	0.630	0.429
No	25 (80.6)	119 (86.3)	-23.8-35.2		
**Control contacts:**	N = 26	N = 38			
Yes	7 (26.9)	11 (28.9)	-45.8-41.8	0.032	0.859
No	19 (73.1)	27 (71.1)	-41- 45.8		

## Discussion

The tuberculosis patients in the study and the control groups at baseline have comparable socio-demographic variables as shown in Table 1. Both groups have no significant differences in their mean age, gender, marital status, education, and occupation. They thus differ only in the expose to health education intervention and are matched for comparison. The baseline knowledge of the cause of tuberculosis was poor and differed significantly (P = 0.000) among the two groups. Both groups still have the erroneous and socio-cultural beliefs that TB can be caused by evil spirits and poisoning See Table 2. The implication of this is that appropriate treatment will not be sought on time, thereby leading to delay in diagnosis and treatment of tuberculosis. The observed differences in the two groups’ knowledge of the cause of tuberculosis may be as a result of different prior exposures, experiences and inadequate routine TB health education in the respective TB centres. The differences could also serve as an indicator of the measure of the effectiveness of routine TB health education in the two TB centres. However, since TB health education is routinely given at both the study and control TB units, the baseline differences in knowledge could also be a result of inherent differences in the individual’s ability to learn, a factor that only becomes obvious at the baseline and post-intervention evaluation as in this case. The level of difference in knowledge contributed by the health education intervention became evidenced in the post-intervention analysis. Similar poor knowledge of the cause of tuberculosis as found in this study was reported in a survey study on obstacles for optimal tuberculosis case detection in Primary Health Centers conducted in Sidoarjo district, Indonesia, where 60% of the respondents did not know the cause of tuberculosis [23]. Other survey studies have similarly reported poor knowledge of the cause of TB disease among sufferers [24, 25].

The knowledge of the responding TB patients about the risk factors and mode of transmission of tuberculosis was also poor for both the study and control groups at baseline See Table 2. The result is similar to studies done in Cameroon and Ethiopia, where poor knowledge of the risk factors and mode of transmission was recorded [26, 27]. However, this differs from the finding in a study done in Dar es Salam, Tanzania, where 78.4% of patients had good knowledge on tuberculosis transmission [24]. The Tanzania study however reported poor knowledge of the risk factors of pulmonary tuberculosis among the participating patients as only 38% knew about the risk factors [24]. This poor knowledge would probably aid transmission of tuberculosis in these areas. Lack of adequate health education to the patients on tuberculosis treatment in the study sites may have contributed to their high level of ignorance about the TB disease evidenced in this study and should be corrected. The observed poor knowledge may have also reflected in the findings about contact tracing among the same TB patients in this study.

Respondents in the study group unlike in the control group had good knowledge about the control of tuberculosis transmission. About three quarters of them knew that closing their mouths while coughing, improved natural ventilation, good housing, early diagnosis and treatment, and immunization with BCG were control methods as shown in Table 2. Similar level of good knowledge of TB control mechanism comparable to the findings in this study was strongly reported in the study done in Tanzania [24], but less strongly when compared with the report in the Cameroonian study [27]. This level of good knowledge should be used to encourage TB patients to take their children for BCG immunization, and to promote the use of other control measures, that could help reduce the transmission of tuberculosis in the study area.

Contact tracing, which involves the evaluation of persons who have been in contact with patients having tuberculosis, can be active or passive. In active contact tracing, the health worker regularly visits the family of each TB case and keeps a contact register, while in passive contact tracing, the health worker gives adequate health education to each TB patient in order to stimulate him or her to present any family member with suspect symptoms to the health service [13]. In practice health education of patients in this part of the world is far below adequate, and where done, an informal approach is used, which is unplanned, sporadic and off-handed way of disseminating health information to patients. Patient’s educational progress is not documented, neither reinforced, nor coordinated and depend entirely on the whims and inclinations of the particular health care team [28]. The same half hazard approach to formal patient health education in the hospitals is used in giving TB patients health education on passive contact tracing.

The TB patients in this study who received the formal, targeted, focused and planned health education learning experiences were expected to acquire knowledge and accurate information about tuberculosis disease, and the skills needed to convince and bring their contacts for TB screening. The proportion of the TB patients in both the study and control groups at baseline that were aware of contact tracing prior to this study was below 20% in each group. The low awareness of contact tracing found in this study is similar to the observation in a survey study done in Malawi where, although 40% of the patients with smear positive pulmonary TB had young children living with them at home, but only 21% were aware of the need for childhood screening [17]. However, the study group had a tremendous improvement in their awareness of the importance and need for contact tracing post-intervention when compared with the control group. There was statistically significant difference in the awareness of contact tracing among the study group pre-and-post health education intervention, unlike in the control group (X^2^ = 158.4, DF = 1, p = 0.000; 95% CI: 15.8-82.2 verse X^2^ = 3.31, DF = 1, p = 0.069; 95% CI: -9.9-24.7). The gains achieved through the health education intervention in this regard are in line with the survey findings stating that the successes achieved in the control of tuberculosis worldwide especially in Nigeria and many other African countries were attributed to many programme elements such as directly observed treatment short course (DOTS), health education, reminders, contact tracing, TB screening, and defaulters tracing [8–10].

The knowledge of the groups about contact tracing was assessed at baseline and post-intervention. The study and the control groups had poor knowledge of the meaning and the essence of contact tracing at baseline. The observed difference was statistically significant (x^2^ = 26.32, df = 1, p = 0.000, 95% CI 7.3-44.2). This difference may not be unconnected to the varying exposures of the two groups to TB health education prior to the intervention. However, enhanced effect of the focused and planned health educational intervention on the knowledge of contact tracing was observed among the TB patients in the study group post-intervention. The knowledge of the study group about contact tracing increased markedly, unlike in the control group. The post-intervention difference in the knowledge of the complete meaning of contact tracing was highly statistically significant ( x^2^ = 147.22, df = 1, p = 0.000, 95% CI 49.3-80.5 ) in favour of the study group. The knowledge of the correct and incorrect meaning of contact tracing also was greatly enhanced among the TB patients in the study group, unlike the control group which maintained the statuesque knowledge. Also the difference in intra or within group comparison of knowledge of contact tracing among the study group was highly statistically significant, unlike the control group (x^2^ = 65.03, df = 1, p = 0.000, 95% CI 23.3-58.5 vs. x2 = 0.16, df = 1, p = 0.686, 95% CI -14.9- 18.3) as shown in Tables 3 and 4. The improvement in the knowledge of contact tracing recorded among the study group gives credence to the expected outcome of health education as stated by the Joint Committee on Health education and Promotion, and the World Health Organization, that it helps improve the individual’s health literacy level as well as providing him with the necessary information and knowledge needed to make informed quality health decisions [21].

The health education intervention on contact tracing was not only aimed at improving knowledge, but also imparting the skill required by the TB patients to convincingly brings their contacts for evaluation and treatment where screening revealed positive result. It also requires commitment to changing personal factors and value system re-orientation such as self-efficacy (perceived ability/competency), attitude (beliefs and values about the outcome of the behaviour), and subjective or social norms, that is beliefs the person held in regard to others expectation of his behaviour. This is the conceptual basis for the designing of the planned health education interventions geared toward persuading the TB patients for behavioural change and giving them skills to be able to convincingly bring their contacts for TB screening [29, 30]. The application of these behavioural change theories as learnt during health education intervention played a significant role in the observed changes in contact tracing practice among the study TB patients post-intervention. At baseline, the percentage of the smear positive TB patients in the study and control group that practice contact tracing and the number of contacts brought for screening was very infinitesimal and insignificant compared to the known infectivity rate Table 5. Over 85% of the study and control TB patients respectively did not bring their TB contacts for screening at baseline (153 vs. 154, X2 = 0.57, p < 0.451, 95% CI 10.7-15.9). Considering the high level of social interaction and intimate relationships going on in our extended family system, one can appreciate the large number of contacts the TB patients would probably make in a day on continuous bases that averts the TB screening exercise. It is estimated that 2–3 persons would be infected by a smear positive case before its detection in developed countries, while it is 4–5 persons in the developing countries because of higher number of close contacts [14]. Among the study group that received the health education, number of TB patients that brought their contacts for screening at baseline, and the actual number of contacts brought post-intervention was very remarkable. The observed differences in the number of TB patients that brought contacts for screening and the actual number of contacts brought for screening among the study and the control groups pre-and -post intervention was highly statistically significant (144 vs. 9, X2 = 134.95, p = 0.000. 95% CI 44.3-74.9) Table 5.

The improvement in the awareness of contact tracing, knowledge of its meaning and purpose, with the overwhelming increase in the number of the TB patients that brought contacts for screening post-intervention in this study have shown that planned health education intervention can be used to increase case detection, thus capable of reducing treatment delay and enhancing TB control mechanism. An educational outreach carried out among nurses in South Africa reported similar increase in case detection after the outreach programme [31]. Treatment delays in sub-Saharan Africa have been additionally attributed to the lack of awareness as reported in some studies [27, 32, 33]. These findings are similarly observed in this our study at base line and among the control group without health education post-intervention. It has also been reported that delay in diagnosis of tuberculosis and commencement of treatment was common in Nigeria and other countries [24, 34]. The study in Lagos reported that 81% of patients delayed going to a health facility had low level of knowledge about their disease [34]. Thus, the low level of knowledge about TB disease as observed in this study could lead to delay in seeking for treatment among the TB patients in these centres. Longer delays have been associated with worse clinical outcomes, greater disease transmission and risk of death [31, 33].

Tuberculosis contacts brought for screening by the TB patients in the study and control group approximately have equal male and female ratio. Majority of the contacts were less than 10 years of age, single, students and live with the patients and are mostly siblings of the TB patients Table 6. These socio-demographic findings are very significant and similar to findings from other studies within and outside the country [20, 34, 35]. High risk populations include preschool and school populations in communities with a TB incidence of >30/100,000/year as in most communities in Africa [22]. A survey study done on tuberculosis contact tracing among children and adolescents in Brazil, revealed that 92.4% of them had household contacts, 66.5% of the contacts were the child’s parents [20]. In analysis of a nationwide case finding and treatment outcomes of childhood tuberculosis in Malawi, it was found that young children from households where one or both parents have smear positive pulmonary TB are at an increased risk of developing active and disseminated tuberculosis. The study further revealed that 40% of patients with smear –positive TB had young children living at home [17]. A review study of TB contacts by the Center for Disease Control and Prevention in the United States of America between July 1996 and June 1997 showed similar findings regarding the number of household or close contacts with TB. Out of the 6,225 close contacts of TB patients examined, 43% were household contacts, 18% relatives not living in the household, 12% were close co-workers, 9% were leisure contacts while other type of contacts were 18% [18]. The household members and close associates of TB patients comprise a high-risk group for tuberculosis and as such their examination carries greater importance regarding prevention and control of tuberculosis [11]. A survey of TB infection in children who are contacts of immigrants’ TB patients in Netherland similarly revealed a high prevalence of active and latent tuberculosis infection among these children [35].

A systematic review and meta-analysis of 203 published studies out of 9,555 screened, reporting prevalence of TB and TB latent infection, and the annual incidence of TB among contacts of patients with TB was carried out by Fox GJ, Barry SE, Briton WJ, and Marks GB [36]. The results of the 95 studies from the low-and-middle-income settings varied considerably from the findings in the 108 studies from high-income settings. In the low income settings, the prevalence of active TB among all contacts was 3.1% (95% CI 2.2-4.4%), microbiologically proven TB was 1.2% (95% CI 0.9-1.8%), latent infection 51.5% (95% CI 47.1-55.8%), and the prevalence of TB among household contacts was 3.1% (95% CI 2.1-4.5%). Among the high –income countries, the prevalence was 4% (95% CI 1.1-1.8%) among the TB contacts, and 28.1% (95% CI 24.2-32.4%) for latent infection. In our present study, the prevalence of TB among contacts was comparatively higher, both for the study and control groups Table 8. Among the study contacts, the prevalence of TB among the contacts before and after intervention, were 6 (19.4%) and 19 (13.7%) (95% CI -23.8-35.2); and in the control group 7(26.9%) and 11(28.9%), (95% CI -45.8-41.8) respectively. The observed differences in the presence of TB among the contacts of the study and control groups were not statistically significant (P > 0.05). However, there was a statistically significant difference between the study and control groups in the primary outcome measure, that is the number of TB patients that brought their contacts for screening post intervention ( 114 verses 9; 95% CI 44.3-74.9) Table 5. Similarly, in South East Asia, a systematic review and meta-analysis of 11 studies that met the inclusion criteria out of 1087 screened were analyzed to investigate the prevalence of TB infection among child contacts of TB cases [37]. The result showed the prevalence of TB infection among child contacts under 15 years of age was between 24.4 -69.2%, quite higher than the prevalence TB diseases in the region which varied from 3.3% to 5.5%. The prevalence of TB among the contacts in our study as shown in Table 8, both before and after the intervention, falls within the range stated among child contacts in the South East Asian review study Table 8. In either case, the result is an indication for a search of innovative way or novel approaches to enhance contact tracing among TB contacts.

The TB contacts brought for screening both by the study and control groups at baseline and post-intervention had the cardinal TB infection signs and symptoms of cough, fever, weight loss and night sweats in the decreasing order of prevalence. Tuberculin test, chest x-ray and sputum acid fast bacilli (AFB) microscopy results were positive in varying degrees between the contacts Table 7. However, less than a quarter and one-fifth of contacts brought for screening by both the study and control TB groups at baseline and post-intervention respectively were diagnosed and treated as TB case Table 8. The prevalence of TB among the close contacts as found in this study closely compares with an estimated 22% infection rate among people with prolonged, frequent or intense contact with TB cases [1]. However, this differs from the lower confirmed cases of pulmonary tuberculosis found among adult populations in the Iranian provinces [38], but compares favourably with the high prevalence value observed among children contacts of immigrants in the Netherland [35]. The Iranian survey study of 147 close contacts of 34 index TB patients shows that 38.2% had an indurations more than 15 mm considered positive in this study, 33.3% showed abnormal radiological manifestations, with 4.8% confirmed as cases of pulmonary TB [38]. The TB contacts are estimated to be 10 to 60% times more likely to have the disease than the general population, and approximately 10-14% of all notified cases have been detected by contact screening [14]. The slight differences observed in this study regarding the prevalence of TB among TB contacts screened may be a factor of diagnostic criteria, as it concerns the use of chest x-ray, tuberculin test, and sputum AFB. It may also reflect the clinical acumen of the clinicians in interpreting negative test results, in the presence of obvious clinical signs and symptoms especially among children.

Generally, planned and focused health education intervention has been successfully used to improve the knowledge of TB disease and contact tracing skills of the TB patients in a major TB centre in Enugu State. The differences in the primary outcome measures, that is the number of TB patients that brought their contacts for TB screening, and the actual number of contacts brought for screening were statistically significant (p < 0.001) Table 5. Also the difference in the secondary outcome measure, that is the knowledge difference about contact tracing between the study and control groups post-intervention was statistically significant ( p < 0.001) Table 4. The null hypothesis of no significant difference on the knowledge and skills of the study and the control TB patients about contact tracing post-intervention is therefore rejected.

Fidelity of Implementation, Success of and Barriers to Implementing the Intervention.

The intervention was implemented by a team of researchers comprising of the three principal researchers, six nurse educators trained on the content of the health education, research protocol, questionnaire contents and administration. Others include three laboratory technologists that performed and read the sputum smear microscopy, and who also received training on the health education content, and two external consultants quite knowledgeable with training on the research protocol acting as observers and evaluators of level of implementation fidelity, monitors and gives feed back at the end of the sessions. The trainers adhered to the essential components of the intervention including the content of the health education, frequency of the intervention, duration of each training session and specific content coverage prescribed for each session. The trainers used printed training guide to ensure strict adherence to the training protocol, and to improve quality of content delivery. The content of the health education were made simple and less complex, and the questionnaire was also simplified and made less ambiguous after pre-testing in a similar setting. The trainees were given training hand outs and materials including the objectives of the training. Feedbacks were requested from trainees after each training period for the benefit of those delivering the intervention so as to improve their facilitation strategies. The content of health education intervention include the causes, signs and symptoms of TB; mode of transmission, spread and control; benefits of early detection , diagnosis, and treatment; dangers of untreated cases of TB; fallacies associated with TB; meaning and importance of contact tracing; and the framework for attitudinal and behavioural changes toward community health, and TB patients. Although the participants were highly engaged, motivated, and committed to attending the training sessions, some challenges were still experienced. Some sessions started late because the participants were unable to keep to the time schedule for one personal problem or the other including local transportation delays. Those that missed more than two sessions were considered among the drop outs. Also the quest to start normal daily clinic consultations imposed a little challenge, especially on the time allotted for question and answer sessions for the participants to share their views and get clarification on intriguing issues and challenges.

### Limitations and generalization of the findings

Standard classical experimental study, involving random selection of TB units from all the states of the Nigerian federation would have given better internal and external validity, thus more generalized than quasi-experimental design. However, the careful follow up of the scientific methodology involved in quasi-experimental studies, has made the generalization of the findings of this study possible to populations and settings, treatment and measurement variables. The biggest TB centers studied sub serves the largest number of patients, and takes referral from all over Nigeria, more especially from the south eastern states. A multistage sampling method was used to ensure equal chances of selection. At the patient level, all the patients that were present during the eight weeks long period of recruitment and gave orally informed consents were included in the study. This further expands the scope and coverage of the study. Also a calculated sample size was used based on standard power and a prevalence value from previous studies, thus making the sample a representative sample of all the sputum positive TB patients. Statistical calculations for significant differences were also done to close the possible gaps or methodological limitations where present. Drops outs, incomplete or improperly filled questionnaires were equally discarded in both the study and control groups. The small percentage attrition would not have constituted internal validity problem. The study was done in a natural or real life setting, thus boosting the external validity. All these measures assure that external validity, including population and ecological validities were attained, further increasing the strength of the experimental design. The use of separate study and control groups gives better internal validity, enhancing the principles of cause and effect relationships. Thus, this study can be generalized to settings beyond the study site. However, careful application of the strategies employed and the findings of this study is necessary according to local context in view of the contextual factors in public health interventions as it relates to policies and implementations.

## Conclusion

Focused and targeted health education intervention has been successfully used in this study to strengthen contact tracing knowledge and skills of TB patients to bring their contacts for screening at the major TB centre in Enugu State, Nigeria. At baseline, the awareness, knowledge, and practice of bringing contacts for screening among the TB patients were grossly poor. Post intervention, there was significant improvement in the number of TB contacts brought for screening among the study group, unlike in the control group. These finding have great implication on TB case management, and invariably on the global goal of reducing TB prevalence and death rates by 50% relative to 1990 level by 2015, and eliminating TB as a public health problem by 2050. Since TB has a global burden and of global public health importance, there is need for novel strategies such as the one applied in this research to impact skills and improve knowledge of contact tracing among TB patients, that are apparently acting as both primary and secondary source of transmission of TB. By so doing, the TB patients would become partners in progress toward elimination of TB as a public health problem by 2050. Policy is hereby advocated on the use of formal, planned, coordinated, and participatory intensive health education intervention to improve the contact tracing skills of TB patients at the major TB centres, especially in the low and middle income countries with the larger percentage burden of TB disease.

## Authors’ information

Osa-eloka C Ekwueme is a Consultant Public Health Physician with the University of Nigeria Teaching Hospital Enugu; a Fellow of the National Postgraduate Medical College of Nigeria and a Master’s degree holder of the University of Nigeria. He is also a senior lecturer and currently the Coordinator of the Master of Public Health programme of the University of Nigeria. His subspecialties of interest include Health System Management, Medical Sociology and Community Mental Health. He has published so many articles in these areas.

Babatunde Omotowo is a Consultant Public Health Physician with the University of Nigeria Teaching Hospital Enugu; a Fellow of the West Africa College Physicians and holds a Master’s degree of the University of Nigeria. He is also a senior lecturer with the same university of Nigeria. His subspecialties of interest include Epidemiology and Primary Health care.

Agwuna K.K is a senior consultant and senior lecturer in the Department of Radiology, University of Nigeria Enugu Campus. He is a Fellow of the West African College of Surgeons and International College of Surgeons. He was a onetime president of Nigeria Medical Association, Enugu Branch. He had held several committee posts in the university. He has also published in several reputable journals and had presented several research works in both national and international conferences.
